# Challenges of Using Whole-Cell Bioreporter for Assessment of Heavy Metal Bioavailability in Soil/Sediment

**DOI:** 10.3390/bios15040260

**Published:** 2025-04-18

**Authors:** Shanshan Bai, Zhipeng Liu, Jiazhi Xu, Yongshuo Li, Zirun Zhang, Zefeng Huang, Williamson Gustave, Boling Li, Xiaokai Zhang, Feng He

**Affiliations:** 1 Institute of Environmental Processes and Pollution Control, School of Environment and Ecology, Jiangnan University, Wuxi 214122, China; 2 School of Chemistry, Environmental & Life Sciences, University of the Bahamas, Nassau 4912, Bahamas; williamson.gustave@ub.edu.bs; 3 School of Environmental Science and Engineering, Suzhou University of Science and Technology, Suzhou 215123, China; boling.li@usts.edu.cn; 4 Meadows Center for Water and the Environment, Texas State University, San Marcos, TX 78666, USA

**Keywords:** biosensor, bioluminescence, soil pollution, environmental risk assessment, signal transmission

## Abstract

Soil and sediment contamination with heavy metals (HMs) is a critical environmental issue, posing significant risks to both ecosystems and human health. Whole-cell bioreporter (WCB) technology offers a promising alternative to traditional detection techniques due to its ability to rapidly assess the bioavailability of pollutants. Specifically, lights-on WCBs quantify pollutant bioavailability by measuring bioluminescence or fluorescence in response to pollutant exposure, demonstrating comparable accuracy to traditional methods for quantitative pollutant detection. However, when applied to soil and sediment, the signal intensity directly measured by WCBs is often attenuated due to interference from solid particles, leading to the underestimation of bioavailability. Currently, no standardized method exists to correct for this signal attenuation. This review provides a critical analysis of the benefits and limitations of traditional detection methods and WCB technology in assessing HM bioavailability in soil and sediment. Based on the approaches used to address WCB signal attenuation, correction methods are categorized into four types: the assumed negligible method, the non-inducible luminescent control method, the addition of a standard to a reference soil, and a pre-exposure bioreporter. We provide a comprehensive analysis of each method’s applicability, benefits, and limitations. Lastly, potential future directions for advancing WCB technology are proposed. This review seeks to establish a theoretical foundation for researchers and environmental professionals utilizing WCB technology for pollutant bioavailability assessment in soil and sediment.

## 1. Introduction

Heavy metal (HM) pollution represents a critical global challenge that significantly endangers environmental quality, human health, and food security [1,2,3]. While HMs occur naturally in the Earth’s crust and contribute to background concentrations in soils, rocks, sediments, and water bodies [4,5], their levels in terrestrial and aquatic ecosystems have escalated sharply in recent years because of human activities. The severity and geographical spread of HM contamination in soil and water have been steadily increasing [6,7]. Major sources of HM pollution include wastewater irrigation, HM-containing pesticides and fertilizers, lead-based paints, mine tailings, coal combustion residues, and improper waste disposal [4,8,9]. These pollutants are often transported into water bodies via surface runoff, accumulating in sediments [10,11]. Sediments are a major host of incoming metal ions [12]. Thus, soil and sediment act as major sinks for HMs, with contamination levels closely linked to food and water safety [2].

Over the past half-century, over 30,000 tons of cadmium (Cd) and 800,000 tons of lead (Pb) have reportedly been discharged into the environment globally, with the majority accumulating in soil and causing severe environmental issues [7]. About 10 million hectares of China’s entire cultivated land—more than 8% of the nation’s total cultivated land—show signs of HM contamination. This contamination directly reduces annual food production by an estimated 10 billion kilograms [13,14]. Similarly, in Russia, 1.7% of surveyed land is classified as “dangerous”, while 9.1% is deemed “moderately dangerous” due to HM pollution [15]. Another major environmental problem is the presence of HMs in sediments. According to the European Environment Agency (EEA), between 75% and 96% of European seas remain contaminated with elevated concentrations of HMs [16]. In China, studies on the alluvial deposits of the Qiantang, Jiaojiang, and Yangtze rivers indicate mild to moderate contamination, with the biggest ecological concern being Cd, followed by mercury (Hg) [17]. In Suzhou City, the average concentrations of Cd, copper (Cu), chromium (Cr), arsenic (As), nickel (Ni), Pb, and zinc (Zn) in river sediments exceed the GB 15618-2018 standard [18]. Consequently, the detection, monitoring, and risk assessment of HMs in soil and sediment are crucial for promoting sustainable environmental development.

Inductively coupled plasma–mass spectrometry (ICP-MS) has become a common approach for detecting HMs; however, its application is limited by the high cost of instrumentation [13,19]. Moreover, traditional chemical detection methods primarily concentrate on determining the total concentration of HMs, which can lead to an overestimation of potential risks. This overestimation often results in unnecessary and expensive soil remediation efforts [20,21]. Such methods fail to adequately represent the bioavailability of HMs, which corresponds to their actual toxic concentrations [3]. Recognizing this limitation, the United States Environmental Protection Agency (USEPA) has determined that bioavailability is a critical parameter for assessing pollutant risk [22]. The rapid and accurate assessment of HM bioavailability in soils and sediments is now a key focus in evaluating the environmental and health risks linked to HM contamination. In response to the global challenge of HM pollution and the shortcomings of traditional detection methods, WCB technology has emerged as a novel, efficient solution for the detection and risk assessment of HM pollution [23].

WCBs are commonly designed and developed to assess the bioavailability and toxicity of environmental contaminants by employing bacteria, fungi, algae, or animal cells as host strains [24]. Lights-on WCBs integrate bioreporter molecules with bioluminescent, fluorescent, or colorimetric signaling elements, enabling the detection of chemical contaminants, their bioavailability, and their toxic effects on living systems [25]. These WCBs offer several advantages, including a straightforward detection process, low detection limits, rapid response times, and cost-effectiveness. They are highly suitable for assessing pollutant bioavailability and conducting large-scale screening for HM contamination [26]. However, detecting contaminants in soil or sediment samples presents greater challenges than pollutants in aqueous phases. The solid particles in soil and sediment can significantly interfere with the optical signal generated by the WCB, thereby weakening the measured signal and complicating the detection process [27].

Currently, there is no standardized method for correcting the attenuation of the luminous signal in WCBs. This article offers an extensive overview of various methods for assessing the bioavailability of HMs in soil and sediment. The advantages and limitations of these methods are critically compared and analyzed, with particular emphasis on approaches for correcting optical signal attenuation during the detection of HMs using lights-on WCBs. This review aims to provide a valuable reference for researchers and environmental practitioners interested in leveraging WCB technology to evaluate the bioavailability of HMs in soil and sediment samples.

## 2. Comparative Methods for the Detection of HM Bioavailability in Soil/Sediment

In the field of environmental science, bioavailability is the fraction of a pollutant that is accessible for uptake and accumulation by biota [28]. Based on their detection principles, methods for assessing bioavailability can be categorized into chemical and biological approaches [16,19,29]. This article compares key chemical methods, such as the extraction of chemical reagents and the diffusive gradients in thin films (DGT), with representative biological methods, including the indicative plant method, the indicator animal method, and WCB technology. Additionally, we provide a detailed synthesis and analysis of the advantages and limitations of these methods, their applicability, and the comparability of their results.

### 2.1. Chemical Method

Existing studies indicate that the bioavailability of HMs is greatly impacted by their chemical speciation in addition to their total concentration [30]. The chemical speciation of HMs largely determines their potential to be utilized by living organisms, thereby defining their bioavailability. Common chemical analysis methods for determining HM bioavailability include chemical reagent extraction and DGT [19,21].

Chemical extraction is the most widely used method for determining the bioavailability of HMs in soil and sediment [16]. Chemical extractants react with specific forms of HMs to separate distinct fractions. In 1979, Tessier introduced a five-step sequential extraction method, classifying HMs into exchangeable, carbonate-bound, iron- and manganese oxide-bound, organic-bound, and residual states [31]. However, this method suffers from low recovery rates, poor reproducibility, and challenges in verifying reliability due to the absence of standard reference materials [32]. To overcome these restrictions, the European Union Agency for Material Standards (BCR) developed the BCR sequential extraction method, which categorizes HMs into weak-acid-extractable, reducible, oxidizable, and residual fractions [33]. This approach has since become one of the most popular techniques for analyzing HM speciation [34]. Notably, HMs in the weak-acid-extractable fraction are highly soluble, making them bioavailable and posing significant ecological risks [35]. Despite their widespread use, chemical extraction methods primarily focus on the equilibrium distribution of HMs and fail to account for the actual binding modes and strengths between HMs and solid particulate fractions in soil and sediment. Additionally, they overlook HMs’ dynamic interactions and equilibrium states in environmental systems [34,36].

The DGT technique offers an alternative, in situ approach for measuring HM bioavailability. According to Fick’s first law of diffusion, DGT is regarded as a pioneering tool for environmental sample monitoring and is widely recognized for its reliability in assessing HM bioavailability [37,38]. A DGT device is composed of two primary elements: diffusion and binding layers. It includes a dynamic binding phase that may quickly capture unstable and free HMs, simulating the diffusion processes involved in plant roots absorbing metal ions [39]. As a result, DGT is considered more trustworthy than traditional chemical extraction methods for predicting HM speciation in environmental samples [38]. DGT is used extensively for in situ measurements of HM speciation [40,41,42,43]. However, DGT is not without limitations. The preparation of diffusion and adsorption gels is complex, the selection of capture agents is limited, and the method is prone to errors in measurement [39,44].

### 2.2. Indicative Plant/Animal Method

**Figure 1:**
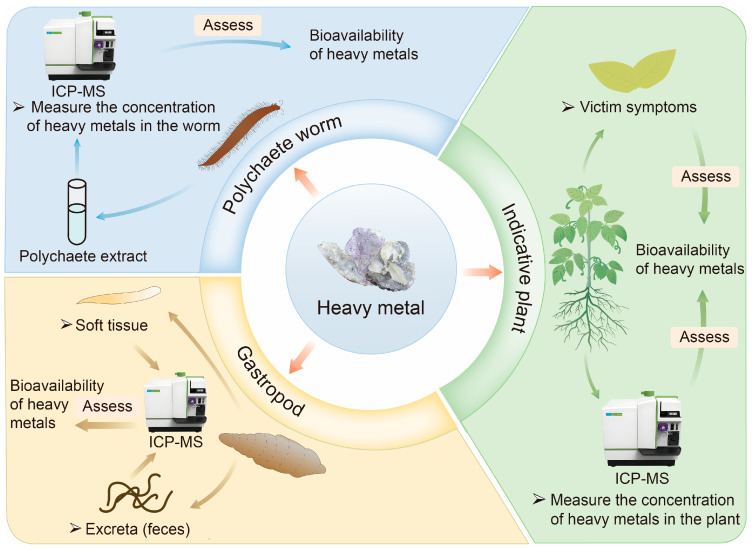
Commonly used biological methods for assessing the bioavailability of HMs in soil/sediment.

Indicator plant and animal detection is an indirect method for assessing HM bioavailability in contaminated soil and sediment. Instead of directly analyzing the soil or sediment, this approach evaluates bioavailability by examining the responses of organisms inhabiting these environments [16]. It leverages the capability of certain organisms to take up and accumulate HMs, using their growth conditions and physiological indicators as biological metrics to assess HM bioavailability [29]. Common methods include the indicative plant, gastropod, and polychaete worm methods, as illustrated in Figure 1 [16,45].

Indicative plant detection is an economical and simple method for assessing the bioavailability of HMs in contaminated soil [46]. Plants such as microalgae, macroalgae, corn, and maize are commonly used to evaluate the deposition, accumulation, and distribution of metal pollutants in soil and water [47,48]. Bioavailability is typically studied by examining plant damage symptoms, including changes in root, stem, and leaf color or morphology, as well as variations in leaf pigment and nitrogen content [49]. Alternatively, bioavailability can be assessed by directly measuring HM concentrations in plant tissues [50]. However, indicator plants are often classified as accumulators, characterized by their ability to concentrate HMs at low environmental concentrations. At higher concentrations, the accumulation factor decreases [51]. Some plants exhibit a repulsive effect, maintaining low internal metal concentrations until regulatory mechanisms fail under extreme environmental metal levels. The long growth period of plants limits the utility of this method, making it more suitable as a supplementary assay for assessing HM bioavailability [49].

Indicator animal assays are another indirect approach, commonly employing organisms such as polychaete worms and gastropods to evaluate the bioavailability of HMs in wetlands and sediment [52]. The primary reason is their sensitivity to changes in soil conditions and their ability to directly reflect the bioavailability of heavy metals [53,54,55]. Polychaetes ingest soil particles and organic matter, coming into direct contact with heavy metals. The accumulation levels of heavy metals in their bodies can indicate the bioavailability of heavy metals [53]. Many benthic organisms, particularly polychaete worms, interact closely with sediments and ingest subsurface layers, which may contain toxic substances [56]. *Hediste diversicolor* (*H. diversicolor*), a polychaete worm that feeds on sediment, is frequently used in such studies. The bioavailability of HMs in sediments can be determined by measuring metal accumulation in sediment samples and the tissues of *H. diversicolor* [45]. Similarly, gastropods live on the soil surface or in the upper layers, indirectly absorbing heavy metals by feeding on plant residues and microorganisms. Their behavior and physiological responses can serve as early warning signals for heavy metal pollution [54]. Gastropods are assessed using detection processes analogous to those for polychaete worms. Their ease of capture and high responsiveness under laboratory conditions make both polychaete worms and gastropods valuable tools in ecotoxicological research [56,57]. However, culturing these organisms is labor-intensive, and the detection process can be time-consuming.

### 2.3. WCB Technology

#### 2.3.1. Detection Mechanisms

WCBs are composed of prokaryotic or eukaryotic cells and are designed as living sensors to specifically target priority pollutants and chemicals of toxicological significance in the environment [24]. A WCB comprises three main components: the sensor, the genetic logic circuit, and the actuator [58]. The sensor element responds to the target compound, thereby triggering a series of biological reactions [59]. The genetic logic circuit connects the sensor and the actuator, converting these biological responses into a detectable signal that is proportional to the analyte concentration [60]. WCBs have proven effective in detecting the bioavailability of HMs in soil and sediment, and they are categorized into Class I, Class II, and Class III types [61,62]. Classes I and II, collectively called “lights-on” WCBs, produce bioluminescent signals through reporter gene expression when the cell’s promoter detects environmental pollutants or cellular stress [25]. Class I WCBs exhibit a high degree of specificity towards certain chemical substances, enabling them to accurately identify and respond to target pollutants [26]. Class II WCBs react to a variety of stress factors. Their response is semi-specific; thus, they are unable to accurately determine the bioavailable concentrations of multiple heavy metals [3]. Class III WCBs are known as “lights-off” types, where exposure to toxic chemicals suppresses reporter gene expression, resulting in reduced bioluminescence [3]. The capacity of Class I WCBs to directly react to quantities of pollutant bioavailability has increasingly attracted attention [63]. Numerous studies have demonstrated the utility of Class I WCBs for quantitatively detecting pollutant bioavailability in environmental samples, including HMs in soil and sediment [61,64,65]. For example, Ivask et al. [66] attached recombinant luminous Hg and As sensor bacteria encapsulated in alginate to optical fibers to create a WCB system. This system was tested on 10 natural samples of soil and sediment from the Aznalcóllar mine in Spain. It was found that the bioavailable fractions of As and Hg were 0.87% and 0.2%, respectively. Similarly, Wu et al. [67] developed Bio-DGT detection technology by incorporating *Acinetobacter baylyi ADPWH_recA* into the DGT framework. The resulting Bio-DGT assay achieved a sensitivity of 0.01 mg/L with stable performance after 30 days of storage. Table 1 summarizes representative studies utilizing Class I WCBs for the detection of HM bioavailability in soil and sediment. Using Class I WCBs to assess the bioavailability of HMs presents many benefits, including rapid responses, enhanced sensitivity, minimal sample preparation, high specificity, and broad applicability. This makes WCBs an efficient, affordable, and user-friendly technology for environmental monitoring [68,69]. In addition, Song et al. [70] compared the results of BCR extraction and WCB detection. The study found that the extraction rates of Cr^6+^ and Pb^2+^ by BCR were 41.1% and 8.3%, respectively, which are relatively close to the results achieved by using WCBs (37.1% and 13.0%). In comparison to chemical methods, the WCB method tends to be faster and more convenient, and the results reflect the true bioavailability.

#### 2.3.2. Factors Influencing the Detection Performance of WCBs

The performance metrics of WCBs in detecting heavy metals, including sensitivity, precision, detection limits, and error margins, are influenced by a variety of factors, such as cell viability, the environmental medium, and the synergistic interactions of heavy metals in environmental matrices [69,77,78,79,80].

Cell viability is one of the key factors affecting the performance of WCBs, which is primarily influenced by cell culture conditions and cell immobilization methods [78,81,82]. Cell culture conditions encompass aspects such as the culture medium, temperature, and duration. Given that most WCBs utilize similar types of microbial strains, their basic culture conditions share certain commonalities [66,70,71,83,84]. Under optimal culture conditions, the detection performance of these strains can generally be stably maintained. In addition, adding certain substances can further enhance the detection capabilities of WCBs. For instance, Pang et al. [81] demonstrated that by incorporating a specific concentration of propylsulfonic acid buffer into the culture system, WCBs could accurately measure the Cu content of samples with a pH range of 0.87 to 12.84, significantly expanding their applicable pH range. This example illustrates that optimizing culture conditions not only improves the viability of WCBs but also enhances their performance in subsequent detection processes.

In addition to the impact of the WCB’s own detection capabilities, the complexity of the environmental medium also has an impact on their detection performance. Soil and sediment samples, characterized by their highly complex matrices, contain numerous potential interfering components, which typically restrict the assessment of pollutant toxicity to biological organisms to the analysis of single pollutants under strictly controlled experimental conditions [85]. In practical applications, when WCBs are exposed to such complex environments, the overall toxic effects of mixed pollutants are difficult to accurately predict through the simple summation of the toxic effects of individual pollutants due to the intricate interactions among multiple pollutants. Preston et al. [80] developed a WCB to detect the biological toxicity of combinations of Zn with Cu, Zn with Cd, and Cu with Cd in an aqueous solution. They found that these heavy metal combinations exhibited significant synergistic toxicity towards *E. coli*. Even though not all common heavy metal combinations were included, the interactions between Zn, Cu, and Cd were sufficient to demonstrate the potential for strong mutual influences among different heavy metals, which may in turn affect the accuracy of the WCB. Currently, despite the widespread application of WCBs in assessing heavy metal bioavailability, many studies still neglect the impact of complex combinations and interactions among heavy metals on detection results [71,73,86,87]. Therefore, to ensure the accuracy of WCB detection results, it is necessary to conduct more detailed research on the toxic interaction effects among various heavy metal combinations in order to avoid deviations between detection results and actual concentrations.

#### 2.3.3. Recent Technological Developments

As technology progresses, the application of WCBs in the environmental field is becoming increasingly extensive. Many new trends and innovations are emerging. They are beginning to develop in the direction of intelligent modular miniaturization.

In recent years, WCBs have gained significant improvements in portability and efficiency and are widely used in the field of microfluidics. Microfluidics enables the miniaturization of analytical equipment by integrating a series of operating units, such as sample pretreatment, reaction, separation, and detection, on a chip of a few square centimeters [88]. The WCB uses the microchannel of the microfluidic chip to accurately control the sample flow from nanoliters to microliters. By integrating this flow control with the inherent functionality of the WCB, highly sensitive and highly selective detection of target analytes in water samples is achieved. The process typically works through specialized recognition interactions with specific biological components and target analytes, such as enzymes, antibodies, antigens, and DNA probes [89]. Cheng et al. [90] constructed an innovative reusable smartphone-based mobile fluorescence WCB for the quantitative detection of bisphenol A and norfloxacin by integrating a miniaturized full-fiber optic and microfluidic system with a smartphone. Miniaturization technology has made WCBs portable while significantly increasing their sensitivity and specificity. This makes WCBs a powerful tool for rapid detection in the field. Its innovative design makes the detection process simpler and more efficient, and the cost is low, making it easy to apply to environmental monitoring, food safety detection, and other areas, providing a new solution for real-time detection and early warning. However, this technology still faces issues. In terms of sensor selectivity, distinguishing between compounds with similar structures and chemical interactions remains a challenge. There are interfering contaminants in the actual water sample that affect the accuracy of the test results. Some microfluidic sensors have to be combined with large instruments, which limits their portability and applicability. In addition, contaminants in water are often mixtures of several compounds, requiring multi-target analysis, and microfluidic sensors are still in their infancy for complex mixture analysis [88].

With the introduction of synthetic biology and artificial intelligence technologies, intelligent WCBs can rapidly process and analyze complex environmental signals. By introducing new standard modules and sophisticated controllers, genetic circuits are being designed using engineering principles for precise signal processing [91]. An intelligent WCB based on synthetic biology is expected to be able to monitor a wide range of environmental pollutants in real time by designing genetic circuits with automatic calibration and memory storage capabilities. Wang et al. [92] designed an AND logic gate using Hrp manipulators to construct an *E. coli* WCB. The WCB can simultaneously detect and integrate three environmental signals, As^3+^, Hg^2+^, and Cu^2+^, using its intrinsic two-component signal pathways or synthetic signal sensors derived from other bacteria integrated with a cell–cell communication module [92]. Intelligent WCBs can not only respond quickly to environmental changes but also maintain high sensitivity and specificity in complex environments, providing powerful technical support in the fields of environmental monitoring, disease diagnosis, and biosecurity. This indicates that the field of environmental monitoring will enter a new stage of greater intelligence and precision in the future. However, intelligent WCBs still face challenges such as long detection times and low automation. An effective way to address these challenges is to expand the library of modules with more functionalities. The emergence of artificial intelligence, cloud computing, and genomic big data will also bring more opportunities for improvement [93].

## 3. Methods Used for the Correction of Soil/Sediment Blocking of WCB Optical Signals

**Figure 2:**
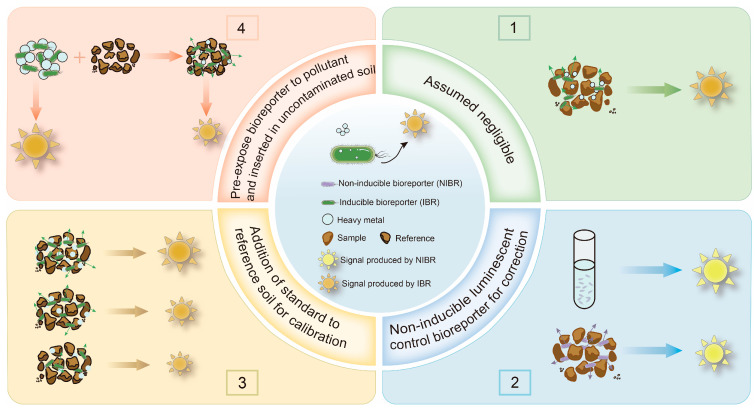
Schematic diagram of the approach to measuring the bioavailability of HMs in soil and sediment using a WCB that generates an optical signal.

As mentioned above, solid particles in soil and sediment samples can attenuate the optical signal transmission of WCBs [94]. Many studies have overlooked this attenuation effect, leading to significant errors in bioavailability measurements, sometimes by an order of magnitude [27]. To address this challenge, we reviewed methods for using WCBs to detect the bioavailability of HMs in soil and sediment, as illustrated in Figure 2. These methods can be categorized into four main approaches: the assumed negligible method, the non-inducible luminescent control method, the addition of a standard to a reference soil method, and the pre-exposure bioreporter method. While the assumed negligible method does not account for signal attenuation, the latter three methods incorporate corrections for the impact of solid particles, enabling a more accurate determination of bioavailable HM concentrations [27].

### 3.1. Assumed Negligible Method

#### 3.1.1. Aqueous Extract

The aqueous extract method involves using deionized water as an extraction agent. Dry soil or sediment samples are mixed with deionized water in a specific proportion, shaken, and then centrifuged. The resulting supernatant serves as the water extraction solution [63]. The cultured WCB strain is then directly exposed to the aqueous extract, and the bioluminescent signal is measured using a plate reader [20]. However, when detecting HMs in soil or sediment, this method is limited by the insufficient extraction capacity of deionized water for most HMs, resulting in very low concentrations of bioavailable HMs in the water extraction solution [27,95]. Ivask et al. [95] reported that significant HMs remain adsorbed onto soil particles, with only approximately 0.6% Cd, 1.3% Hg, and 2% Zn being detected in WCB assays using water extracts. Similarly, Lappalainen et al. [96] employed the light-emitting bacterial strain *E. coli* MC1061(pTOO11) to measure Hg in sediment samples. Their results showed no detectable Hg compared to traditional methods for measuring total Hg. These findings suggest that when Class I WCBs are used to detect bioavailable HMs in aqueous extracts, they may significantly underestimate the environmental risks posed by these contaminants.

#### 3.1.2. Suspension

A suspension can be prepared by mixing dry soil or sediment samples with water in a specific proportion and thoroughly oscillating the mixture [63]. Unlike the aqueous extract method, suspension detection allows WCBs to interact directly with sample particles. Studies have shown that the bioavailable Hg content detected in suspensions was higher than in water extracts [96]. Another study reported that bioavailable Cd and Hg concentrations increased significantly in suspension detection, approximately 20 and 30 times higher, respectively, than in aqueous extracts [95]. Direct exposure to particles enables more accurate detection of the bioavailability of HMs, facilitating a better assessment of their environmental and biological impacts. This approach provides a stronger scientific foundation for risk assessment and managing HM pollution.

However, the scattering and reflection of light by solid particles in the suspension can significantly affect the transmission of the WCB’s bioluminescence signal, leading to signal attenuation and reduced measurement accuracy [27,97]. Additionally, the preparation of the suspension, specifically factors such as mixing time, temperature, and duration, can influence the results. For instance, if the suspension is prepared immediately before measurement, increased turbidity may further diminish the luminescence signal [96]. As a result, the direct contact method with uncorrected suspensions is unsuitable for the quantitative detection of HM bioavailability.

### 3.2. Non-Inducible Luminescent Control Method

The non-inducible luminescent control method uses non-induced WCBs (control WCBs), where the transcription of reporter genes is neither induced nor inhibited by pollutants, resulting in a stable optical signal [27,98]. The luminescence signal of the control strain is influenced only by the physicochemical properties of the sample matrix, such as pH and dissolved organic matter. By comparing the luminescence intensity of the control strain in the sample with that in deionized water or a standard buffer, the non-specific effects of sample particles on WCB luminescence can be assessed and corrected. This correction enables a more accurate determination of the target HMs’ induction effect on the specific luminescent strain.

The non-inducible luminescent control method has been used to evaluate the bioavailability of HMs in soil and sediment extensively [99,100]. Hakkila et al. [87] applied WCBs to assess HM bioavailability in twenty sediment samples (including seventeen synthetic and three environmental samples from the Kishon River, Israel). After correction, the bioavailability of mercury in the Kishon River sediment was determined to be 0.09 mg/L compared to the 0.038 mg/L detected through chemical analysis. Similarly, Ivask et al. [100] evaluated the bioavailability of Cd in 110 naturally aged contaminated soils. They found that Cd in the aqueous extract accounted for only 0.13% of the total Cd in the soil. Still, after correction, the bioavailable Cd concentration in suspension increased by approximately 30 times (3.71%) [100].

By directly comparing the signals of control and experimental strains, the non-inducible luminescent control method provides an intuitive estimate of the effects of soil and sediment particles on WCB signals. It is also relatively simple to operate [27,87,98]. However, this method has limitations. For instance, significant differences in optical properties (e.g., luminescent intensity and spectral characteristics) between control and experimental strains can lead to inaccurate corrections. Additionally, variations in sensitivity to environmental conditions (e.g., temperature and pH) between the control and experimental strains may impact the reliability of signal comparison and correction.

### 3.3. Addition of a Standard to a Reference Soil Method

The method of adding a standard to a reference soil involves adding a gradient concentration of an HM solution to uncontaminated control soil or sediment (with HM concentrations below the detection limit). The luminescent signal generated by the WCB in these spiked soil/sediment samples is measured to establish a standard curve. The optical signal produced by the experimental strain is then corrected using this calibration curve to calculate the bioavailable concentration of HMs in the sample [27,94]. For example, Yoon et al. [94] used uncontaminated standard soil as a reference to prepare soil samples containing varying concentrations of As^3+^ (0–100 ng·g^−1^). They measured the fluorescence intensity of the labeled soil using an FS-2 fluorescence spectrophotometer and constructed a standard curve correlating the bioavailable arsenic concentration with the fluorescence induction coefficient.

This method simulates the interaction of pollutants with particulate matter in soil or sediment, providing more realistic data for assessing pollutant bioavailability [27]. Compared to the non-inducible luminescent control method, this approach allows WCBs to interact directly with the soil matrix, offering a more accurate representation of environmental conditions. However, its practical application faces challenges. Identifying reference soil or sediment with properties identical to the experimental samples is difficult. Variations in soil or sediment characteristics, such as texture, organic matter concentration, or pH, affect the binding behavior of HMs to solid particles. If the reference soil/sediment is different from that of the test sample, especially if the soil has significant contact time with the analyte and the contact time is long enough to allow analyte adsorption, the reference soil/sediment is not suitable for bioavailability quantification. This can result in an error of greater than 30% for a comparatively matched reference soil, and more for a mismatched substrate [27]. Consequently, the bioavailability concentration determined in the reference soil may not accurately represent the actual bioavailability concentration of HMs in the contaminated soil.

### 3.4. Pre-Exposure Bioreporter Method

The pre-exposure bioreporter method can be directly used for detecting HMs in soil or sediment [27]. In the pre-exposure method, WCB luminescence is first activated using an appropriate concentration of an HM solution. Once the luminescence signal reaches its maximum, the activated WCB is immediately added to soil or sediment suspensions of varying concentrations, and the luminescence signal is re-measured. This method evaluates the extent to which the suspension attenuates the luminescence signal of the activated WCB.

Zhang et al. [27] investigated the effects of soil, sediment, and biochar suspensions on WCB bioluminescence signal blocking and developed a method to correct the light signal blockage. This correction is crucial for using WCBs to assess the environmental risk of pollutants in certain solids [101]. These samples’ luminescence signal transmission rates were 79%, 60%, and 8%, respectively [102]. For samples with luminescence values lower than those of the control group, potential reasons include either that the actual HM concentration is below the WCB’s detection limit or that the concentration exceeds the WCB’s threshold for normal responses, resulting in signal inhibition. Table 2 provides a detailed comparison of the advantages and disadvantages of the four detection methods discussed and their recommended application scenarios. It can be concluded that the pre-exposure bioreporter method demonstrates the highest level of accuracy and the most extensive generalizability in practical applications when compared to the other three methods, thus establishing it as the most effective method overall.

## 4. Challenges and Outlook

Compared to traditional methods, WCBs offer significant advantages in detecting the bioavailability of HMs in soil and sediment. The four calibration methods discussed in this review address most practical scenarios encountered in field applications. However, due to the complexity of soil composition, future research must focus on improving the detection performance of WCBs while simplifying their operation and extending their lifespan. Additionally, the design and construction of WCBs require advanced genetic engineering techniques, which increase research and development challenges and may raise biosafety concerns. In practical applications, WCBs can also be affected by interference from other environmental substances, potentially compromising their accuracy and selectivity. Furthermore, most WCBs are designed to detect only one or a few specific HMs, each with varying detection limits. A key future challenge lies in creating uniform protocols for WCB calibration to facilitate broader adoption and commercialization.

With the rapid advancement of technologies such as artificial intelligence (AI), the development of new materials, and synthetic biology, the future of WCBs lies in their integration with these cutting-edge innovations. For example, AI-based models could also analyze the nonlinear relationships between WCB signal outputs and HM concentrations, improving prediction accuracy and enabling real-time environmental monitoring. Material science advancements could make WCBs more lightweight and adaptable, allowing real-time monitoring in diverse scenarios, including emergency soil pollution events. This capability would enable rapid quantitative detection, facilitating prompt response plans to minimize pollution impacts. In conclusion, WCBs hold immense potential for soil HM detection and are poised to become indispensable tools in environmental monitoring. Their continued development and integration with emerging technologies could significantly enhance their utility and applicability in addressing global environmental challenges.

## Figures and Tables

**Table 1:** Representative studies on the detection of HM bioavailability using WCBs.

HM	Strain	Reporter Gene	Medium	Concentration Range	Response Time	Reference
Hg^2+^	*Pseudomonas putida*	*phnS-luxCDABE*, *merR-egfp*	Soil	0–24 µg/kg	Several days	[71]
Cr^6+^	*Acinetobacter baylyi* ADPWH_recA	*zntA*	Soil	2 µM	5 h	[72]
Cu^2+^ Hg^2+^ Pb^2+^	*Escherichia coli* (*E. coli*) *MC106*	*matgBFP* *eGFP mCherry*	Soil	0.1–100 μM 0.01–4 μM 0.1–100 μM	12 h	[73]
Cd Cu Zn	*Bacillus megaterium* VR1	*gfp*	Soil	0–10 mg/L 0–20 mg/L 0–100 mg/L	4 h 4 h 7 h	[64]
Hg^2+^ Cd^2+^	*E. coli* TOP10	*eGFP* and *mCherry*	Soil	0–40 μM 0–200 μM	8 h	[74]
As^3+^	*Bacillus subtilis* 168	*gfpmut3a*	Soil/water	0.1–1000 μM	4 h	[75]
Cu^2+^	Not mentioned	Not mentioned	Soil/water/ Living cell	0.459 μM	Not mentioned	[76]

**Table 2:** The advantages, disadvantages, and application scenarios of methods developed for correcting soil/sediment blocking of the WCB’s optical signal.

	Advantages	Disadvantages	Application Scenarios
Assumed negligible method	It is easy to operate, no additional equipment and steps are required, and the cost is low.	The error rate can be very high, up to by an order of magnitude, that it does not accurately reflect the actual biological availability of the pollutant. It is not suitable for scenarios that require high-precision quantitative analysis.	It is suitable for situations where accuracy is not required, such as preliminary screening or the rough estimation of the bioavailability of pollutants.
Non-inducible luminescent control method	The method is relatively simple and easy to implement to evaluate the effect of particles on the signal by using the control biosensing strain of non-induced luminescence. The signal loss caused by particle scattering and reflection can be partially corrected.	If the optical intensity difference between the control and the detected strain is significant, the accuracy of the correction will be affected. When the signal strength varies greatly, a single correction factor may not be applicable.	This method is suitable for occasions where the signal strength changes little and accuracy is required. When the optical properties of the control strain are similar to those of the detection strain, the correction effect is good.
Addition of a standard to reference soil	Adding a standard solution to the control soil simulates the actual detection environment. It can more truly reflect the influence of particles on the signal, making the correction effect better.	If the properties of the control and target soil are very different, or the contact time between the standard solution and the soil is too long, resulting in adsorption, a large error will be generated.	It is suitable for occasions where the soil properties are similar to the target soil, and the contact time between the standard solution and the soil can be controlled. However, it should be noted that the difference between the properties of the control and target soils should not be too large to avoid introducing new errors.
Pre-exposure bioreporter method	By correcting the degree of blockage of the luminescence signal by solid particles, the correction effect is better than the previous two methods, and it is especially suitable for occasions with high accuracy requirements.	The operation is relatively complex, requiring the identification of appropriate concentrations of contaminants to induce the WCB and the need for precise control of experimental conditions.	This method is suitable for applications where high precision is required and complex operations can be performed, such as precise quantitative analysis of specific pollutants in research laboratories. It can correct the influence of particles on the signal and improve the detection accuracy.

## Data Availability

Not applicable.
